# *Chlamydomonas reinhardtii* LHCSR1 and LHCSR3 proteins involved in photoprotective non-photochemical quenching have different quenching efficiency and different carotenoid affinity

**DOI:** 10.1038/s41598-020-78985-w

**Published:** 2020-12-15

**Authors:** Federico Perozeni, Giorgia Beghini, Stefano Cazzaniga, Matteo Ballottari

**Affiliations:** Department of Biotechnology, University of Verona, Strada le Grazie 15, 37134 Verona, Italy

**Keywords:** Antenna complex, Non-photochemical quenching, Biological physics

## Abstract

Microalgae are unicellular photosynthetic organisms considered as potential alternative sources for biomass, biofuels or high value products. However, their limited biomass productivity represents a bottleneck that needs to be overcome to meet the applicative potential of these organisms. One of the domestication targets for improving their productivity is the proper balance between photoprotection and light conversion for carbon fixation. In the model organism for green algae, *Chlamydomonas reinhardtii*, a photoprotective mechanism inducing thermal dissipation of absorbed light energy, called Non-photochemical quenching (NPQ), is activated even at relatively low irradiances, resulting in reduced photosynthetic efficiency. Two pigment binding proteins, LHCSR1 and LHCSR3, were previously reported as the main actors during NPQ induction in *C. reinhardtii*. While previous work characterized in detail the functional properties of LHCSR3, few information is available for the LHCSR1 subunit. Here, we investigated in vitro the functional properties of LHCSR1 and LHCSR3 subunits: despite high sequence identity, the latter resulted as a stronger quencher compared to the former, explaining its predominant role observed in vivo. Pigment analysis, deconvolution of absorption spectra and structural models of LHCSR1 and LHCR3 suggest that different quenching efficiency is related to a different occupancy of L2 carotenoid binding site.

## Introduction

Among photosynthetic organisms, microalgae have high potential with a wide range of applications of their biomass and its derivatives, from food/feed to biofuels and high-value products^1–9^. These unicellular organisms are characterized generally by a higher photosynthetic efficiency compared to higher plants due to the absence of non-photosynthetic tissues^10,11^. However, some bottlenecks need to be faced in order to improve their domestication process: in fact, their photosynthetic efficiency is far lower than the theoretical value, ranging between 1 and 3% on industrial scale^11,12^. In a natural habitat, photosynthetic organisms are exposed to variable light conditions, which can induce photodamage, affecting their photosynthetic efficiency, so they have developed various acclimation mechanisms, such as thermal dissipation of energy absorbed in excess, a process called non-photochemical quenching (NPQ)^10,13–19^. However, this mechanism is activated in excess, especially in controlled photobioreactor conditions, leading to a massive energy dissipation, up to 80% of light absorbed, through heat^10^. In algae, NPQ mechanism is triggered by antenna-like pigment-binding proteins located in thylakoid membranes, called Light Harvesting Complex Stress Related proteins (LHCSRs), which are over-accumulated upon high light exposure^18,20–23^.

In *Chlamydomonas reinhardtii*, LHCSR3 protein is encoded by two almost identical genes *lhcSR3.1/lhcSR3.2* which differ only in the promoter region^18^: this subunit is usually considered as the main quenching subunit involved in NPQ, as evidenced by the strongly reduced NPQ phenotype observed in *npq4* mutant, a *C. reinhardtii* mutant strain depleted of both *lhcSR3* genes^18^. Moreover a clear correlation between LHCSR3 protein accumulation and NPQ properties can be drawn in this model organism, with LHCSR3 increasing its abundance in thylakoid membranes upon acclimation to high light conditions leading to a strong increase in NPQ induction^18,20,24^. LHCSR3 protein function has been characterized in terms of its pigment-binding capacity and quenching properties through in vitro reconstitution by adding pigments to the apoprotein^24–28^. In particular, LHCSR3 was reported to be activated into a quenching form upon protonation of specific glutamate/aspartate residues exposed to the lumen environment^26,27^. Fast spectroscopy analysis proposed multiple mechanisms at the base of the quenching properties of LHCSR3 subunits involving formation of carotenoid radical cation, energy transfer to carotenoid dark states and induction of possible chlorophyll–chlorophyll charge transfer states^28^. A second LHCSR subunit, LHCSR1, is encoded in *C. reinhardtii* by a single gene, being expressed generally to a lower level than LHCSR3^18,29^. LHCSR1 has been reported to partially compensate for LHCSR3 inducing NPQ in *npq4* mutant (knock-out for *lhcSR3.1* and *lhcSR3.2* genes) and being involved in Photosystem I quenching events^16,30^. LHCSR1 has also been reported to be able to quench isolated Photosystem II antenna subunits, called LHCII^31^. However, a direct comparison between the quenching properties of LHCSR1 and LHCSR3 has not been obtained, due to their different accumulation level in vivo. Here, an in vitro study was used to elucidate LHCSR1 quenching capacity by its comparison with LHCSR3: carotenoid-binding properties were investigated by HPLC and spectral deconvolution of the absorption spectra in the 400–520 nm region (Soret region), where both chlorophylls (Chl) and carotenoids absorb, and quenching properties of LHCSR1 protein were then investigated by time resolved fluorescence analysis.

## Results

### Analysis of LHCSR1 and LHCSR3 protein sequences

**Figure 1:**
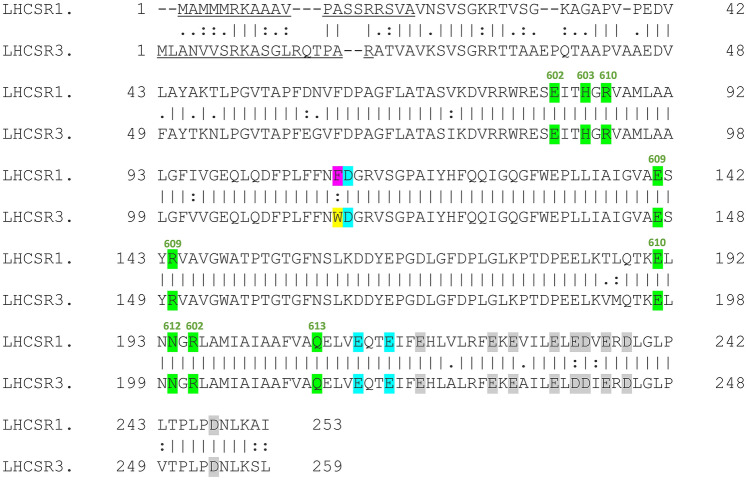
Protein sequence alignment of LHCSR1 and LHCSR3. LHCSR1 and LHCSR3 protein sequences from *C. reinhardtii* were alignment by using Pairwise Sequence Alignment tool. Chloroplast transit peptide predicted by TargetP is underlined. Chlorophyll binding residues are highlighted in green and the coordinated chlorophyll is reported in green for each amino acid. Protonatable residues involved in LHCSR3 protein activation reported in^27^ and in^26^ are respectively highlighted in cyan and grey. Not conserved phenylalanine (F116) residue is highlighted in cyan/yellow.

Alignment of LHCSR1 and LHCSR3 demonstrate a high level of identity between the two subunits (Fig. 1). All the residues previously suggested to be involved in Chlbinding in LHCSR3, as in particular Chls 602, 603, 609, 610, 612, and 613^24,32^ are conserved in LHCSR1. This conservancy is a common feature for Light Harvesting Complexes (LHC), the protein family evolved in eukaryotic organisms involved in the assembly of the external antenna systems of Photosystems^33,34^ with some exceptions: as in the case of LHCSR3^24^, also in LHCSR1 the residues involved in binding Chl 606 and Chl 614 are absent. Other Chls proposed to be bound to LHCSR3 are the Chl 604, 608 and 611 which however are coordinated by a water or lipid molecules, making impossible to prove their presence in both LHCSR proteins in the absence of a detailed 3D protein structure^25,32^. LHCSR subunits were reported to be able to sense the lumenal pH by protonation of specific acidic residues exposed to the lumen, leading to protein activation as a quencher of excitation energy^26,27^: all the glutamate and aspartate residues previously reported to be involved in LHCSR3 activation are conserved in LHCSR1 with the only exception of LHCSR3 D239 residue which is substituted with E (E233) in LHCSR1 (Fig. 1).

**Figure 2:**
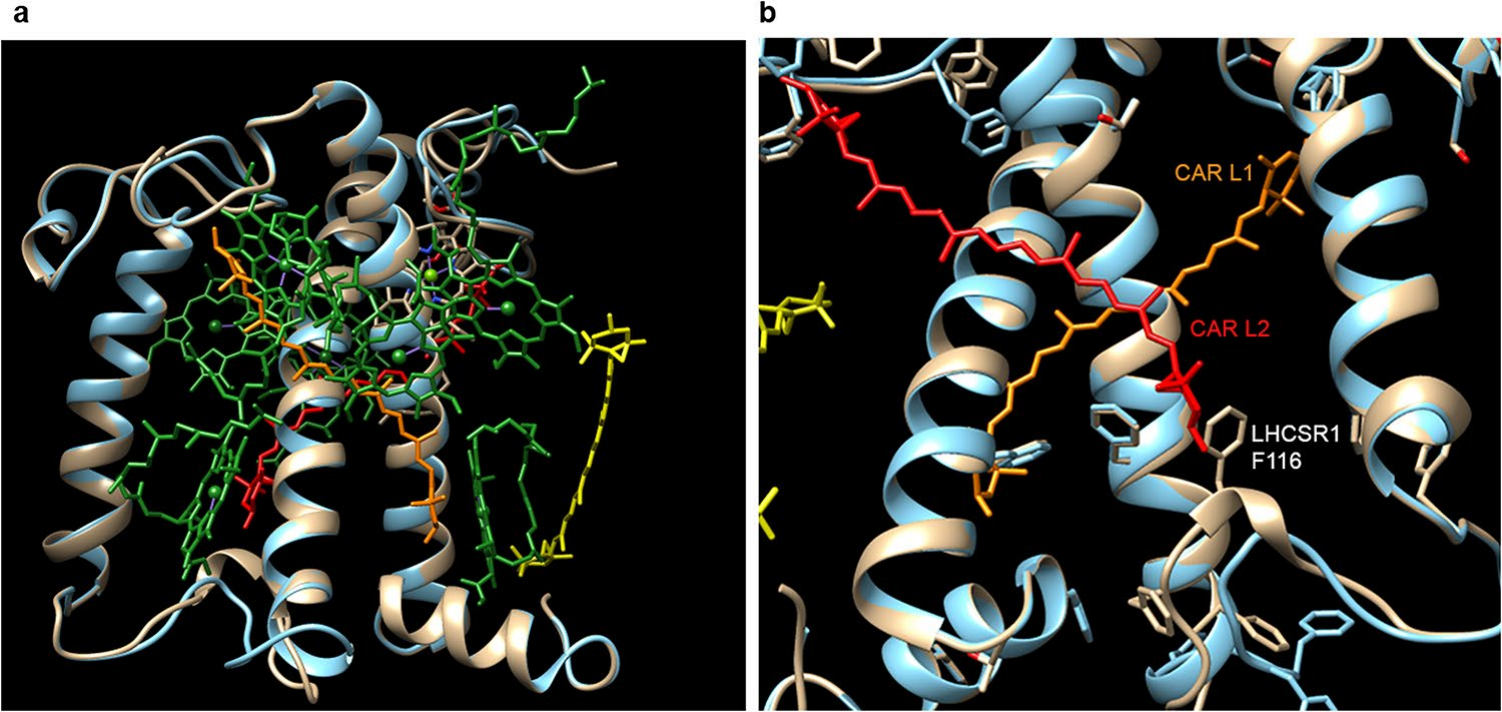
Model structure of LHCSR1 and LHCSR3. (**a**) Structural model of LHCSR1 (grey) and LHCSR3 (blue) obtained by sequence alignment with Lhcb1 (PDB 4lcz.1). (**b**) Enlargement of the L2 carotenoids binding site region with the F116 residue being predicted in LHCSR1 to be in close contact with the terminal ring of carotenoid molecule in L2. Molecular graphics was performed with UCSF Chimera 1.13 software https://www.cgl.ucsf.edu/chimera/

A structural model of both LHCSR1 and LHCSR3 proteins was thus obtained using the previously resolved Lhcb1 structure (PDB 4lcz.1)^35^ including the Chl and carotenoids molecules previously suggested to be present in LHCSR3^25,32^: three carotenoids molecules were in particular included in the LHCSR1 and LHCSR3 protein models being located in the inner sites L1 and L2^36^, while a third more peripheral site was also considered as previously suggested^24^. As reported in Fig. 2 the structural models obtained for LHCSR1 and LHCSR3 were characterized by some minor differences at the N and C terminus and at the level of the loops connecting the different α-helixes. In particular, LHCSR1 structural model is characterized by a peculiar position of F116 residue, which is located in proximity to the terminal ring of the carotenoid in the L2 carotenoid binding site: F116 is not conserved in LHCSR3, where it is substituted by W. The position of F116 in LHCSR1 could influence the occupancy and/or the spectral properties of L2 carotenoid binding site.

### LHCSR1 and LHCSR3 expression and in vitro refolding

**Figure 3:**
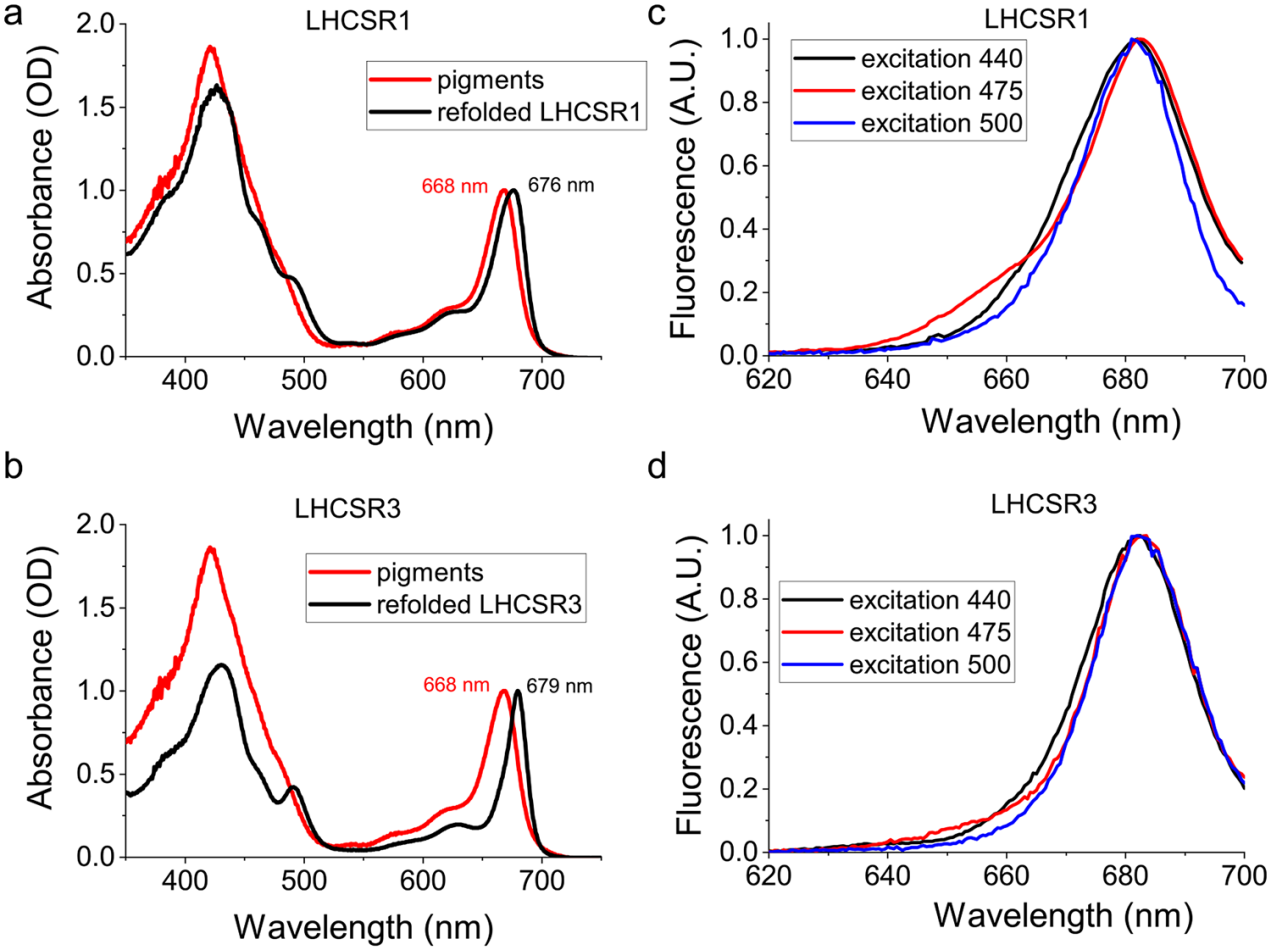
Absorption and fluorescence properties of recombinant LHCSR1 and LHCSR3 refolded in vitro. Absorption spectra were recorded from 350 to 750 nm and compared to the absorption spectra of free pigments in the same detergent solution (Hepes 20 mM, sucrose 0.1 M and beta-dodecyl maltoside 0.03%) (**a**,**b**). Fluorescence emission spectra (**c**,**d**) were measured in the 610–700 nm range upon excitation at 440, 475 and 500 nm. The data herein reported are representative of two independent experiments. The figure was prepared by using OriginPro 2018 software https://www.originlab.com/.

To investigate the role of LHCSR1 in vitro, the encoding *lhcSR1* gene was cloned and expressed in *E. coli* (Supplementary Fig. S1). Purified LHCSR1 apoproteins were then refolded in vitro with pigments, allowing the characterization of its biochemical and spectroscopic properties. The same procedure was performed in the case of LHCRSR3 as previously described^27,32^. Pigments from spinach thylakoids were used for the in vitro LHCSR1 and LHCSR refolding procedure. Indeed, spinach thylakoids share the same pigment composition, even if different relative concentration, as in the case of thylakoids found in C. reinhardtii cells with the exception of loroxanthin, which is present in *C. reinhardtii* but not in vascular plants^37^. However, it was previously reported that LHCSR subunits cannot bind loroxanthin even if present in the pigment mix used to induce in vitro protein refolding^24^. Recombinant refolded holoproteins were investigated in terms of their pigment binding properties. As reported in Table 1 in both cases Chl a, Chl b, lutein, violaxanthin and traces of neoxanthin were detected. The increased selectivity for Chl a rather than Chl b was conserved in both LHCSR1 and LHCSR3 with a slight increase of Chl b content in the former. In both cases more than two carotenoids were found per holocomplex, considering 8 Chl bound per apoprotein: these carotenoids were previously suggested to be bound to the inner carotenoid binding site L1 and L2 and to a third more peripheral site (N1/V1-like carotenoid binding site). Interestingly, the ratio between the amount of lutein and violaxanthin is much higher in LHCSR1 compared to the LHCSR3 case: considering the occupancy of violaxanthin in L2 site previously suggested for LHCSR3, this result might indicate a different composition of this carotenoid binding site. Pigments absorption is tuned by the environment in which they are located: when Chl are in a protein environment, their absorption in the Qy is red shifted compared to pigments in organic solvent or in detergent solution. As reported in Fig. 3, in both LHCSR3 and LHCSR1 holoproteins a clear red shift in the Qy maximum absorption was evident compared to the free pigments case with peaks observed at 679, 676 and 668 nm for LHCSR3, LHCSR1 and free pigments, respectively. In either LHCSR1 or LHCSR3 proteins, it was possible to note that a very low content of Chl b, if any, was present, due to the absence of the peculiar Chl b absorption peaks, usually observed at 475 nm and 650 nm^38^.

**Table 1:** Pigment analysis of LHCSR1 and LHCSR3 refolded recombinant proteins.

	Chl a/Chl b	Chl/Car	Chl tot	Neo	Vio	Lut	Cars	Lut/Vio
LHCSR1	4.10	3.40	8.00	0.11	0.49	1.75	2.35	3.57
LHCSR3	6.31	3.01	8.00	0.03	1.20	1.43	2.66	1.19

Total amount of carotenoids (Cars) and the level of the different xanthophyll were normalized to 8 chlorophyll (Chl tot) content per aproprotein. *Neo* neoxanthin, *Vio* violaxanthin, *Lut* lutein. Errors are below 15% in each case (n = 2).

Correct protein refolding was then investigated measuring fluorescence emission spectra, which allow the evaluation of the possible presence of free pigments in the refolded proteins (Fig. 3). When pigments are bound to an LHC protein, energy transfer among them occurs in the range of ps, until the final emitter, Chl a^25,39^. Thus, upon excitation of different pigments, the resulting fluorescence emission spectra should overlap with the emission spectrum obtained upon excitation of Chl a only. Chl a, Chl b and carotenoids were excited at 440, 475 and 500 nm respectively, and emission spectra obtained were analyzed (Fig. 3). Both LHCSR3 and LHCSR1 proteins where characterized by almost overlapping fluorescence emission spectra obtained at the different excitation wavelengths, demonstrating protein folding and energy connection among pigments bound. Only in the case of Chl b excitation (excitation at 475 nm) the resulting emission spectra showed a partial pigment disconnection being characterized by a small contribution in the 640–660 nm range which could be ascribed to free or loosely bound Chl b.

### Carotenoid composition and carotenoid-binding properties

**Figure 4:**
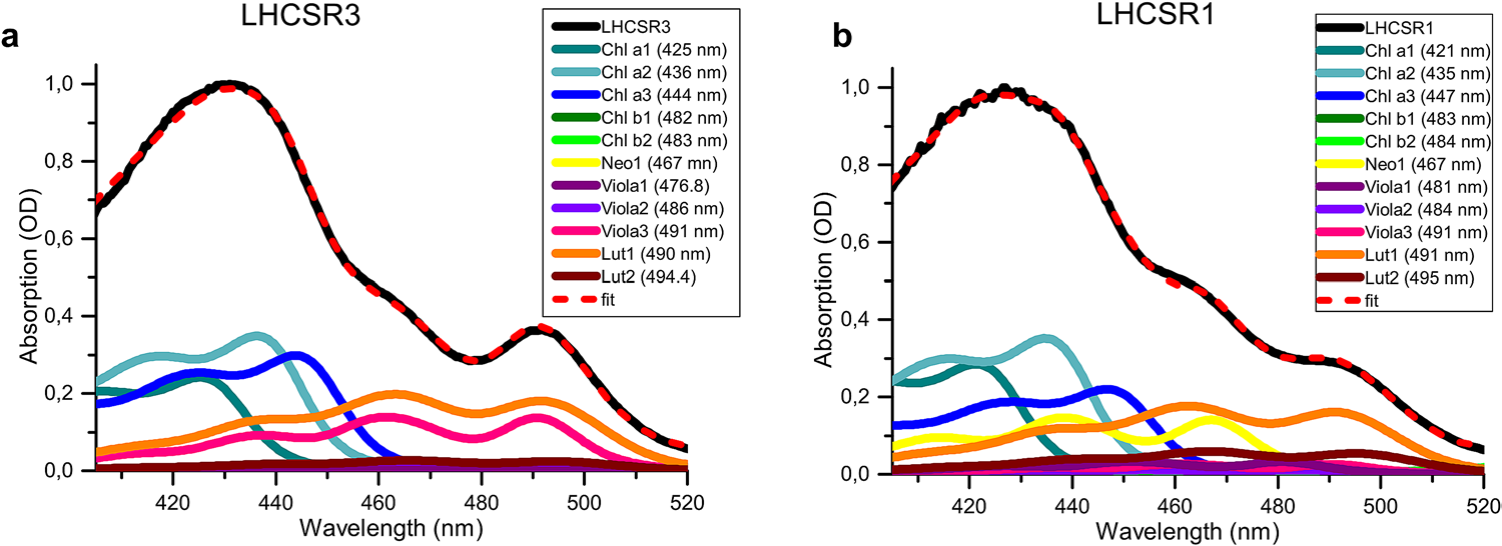
Deconvolution of LHCSR1 and LHCSR3 absorption spectra in the Soret region with chlorophyll and carotenoids spectral forms. Absorption spectra of reconstituted LHCSR3 (**a**) and LHCSR1 (**b**) in the Soret region is represented by the black solid lines. The absorption forms of the single pigments were used to fit the Soret region of the absorption spectra of the complexes accordingly to the constraints posed by different stoichiometry of pigments. Wavelength of the lowest energy absorption peaks in the Soret region are reported for each spectral form in brackets. Spectral deconvolutions of the absorption spectra are shown in red. *Chl a/b* chlorophyll a/b, *Neo* neoxanthin, *Viola* violaxanthin, *Lut* lutein. The figure was prepared by using OriginPro 2018 software https://www.originlab.com/.

Carotenoid absorption is strongly affected by their binding to different sites within LHC proteins^40^. In order to gain information on the multiple occupancy of binding sites by xanthophylls, the absorption spectra in the Soret region (400–520 nm) of refolded LHCSR1 and LHCSR3 were fitted with Chl and carotenoids absorption forms, as previously described^40,41^: transition energies of bounded xanthophylls are indeed strongly affected by the refraction index of the medium and by the ligation to different protein sites^40^. Xanthophylls can be either buried in the protein structure (sites L1, L2) or be more exposed to the solvent (sites V1, N1), resulting differentially shifted compared to their absorption spectra in organic solvent^40,42^. In the case of LHCSR3, the best fitting was obtained with three Chl a, two Chl b, two Lutein, three Violaxanthin and one neoxanthin spectral forms (Fig. 4). The different carotenoid spectral forms could be divided into three groups according to the amplitude of their shifts in the absorption wavelength of the red-most peak in the Soret region with respect to the value in organic solvent. Shifts were of 13 nm, 16–17.8 nm and 8.1 nm, which can be associated to spectral tuning induced by binding to L1, L2 and N1/V1-like carotenoid sites respectively, as for previous analysis in members of the LHC family^42^. According to this model, L1 carotenoid binding site is mainly occupied by lutein (96%) with a small amount of violaxanthin bound (4%). Instead, in the case of L2, 83% of the site is occupied by violaxanthin and 17% by lutein, in agreement with previous findings in the case of other monomeric LHC proteins^33,41^. Traces of neoxanthin and of a third spectral form of violaxanthin, in both cases with a reduced spectral shift compared to the same carotenoids in organic solvent were also found, being assigned to a third more peripheral site, which was previously associated to a V1/N1-like site^24^. In the case of LHCSR1, absorption spectra were similarly fitted by using three Chl a, two Chl b, two lutein, three violaxanthin and one neoxanthin (Fig. 4). Shifts applied to carotenoid spectral forms were similar to those used for LHCSR3, allowing the identification of L1 (with a 11–13.4 nm shift) and L2 (17.2–17.4 nm) sites, which were occupied respectively by lutein (L1 with only 6% of violaxanthin bound) and both lutein and violaxanthin (L2 with 70% lutein and 30% violaxanthin). Interestingly, the composition of L2 site is markedly different in LHCSR1 compared to LHCSR3, where the relative amount of violaxanthin bound is much higher. A third carotenoid binding site was also found in LHCSR1 with a much lower shift compared to carotenoid absorption form in organic solvents being occupied by violaxanthin and neoxanthin absorption form, as in the case of LHCSR3.

### LHCSR1 and LHCSR3 quenching properties

Fluorescence lifetime of LHC proteins is inversely proportional to their quenching activity^43^. C terminus of LHCSR3 protein is known to be responsible for pH-driven conformational change from a light-harvesting to a quenched state^26,44^. To investigate the quenching properties of LHCSR1 protein, compared to the LHCSR3 case, time resolved fluorescence analysis was performed at different pH and detergent concentrations. These analysis allowed to investigate the possibility of LHCSR1 to be differentially quenched depending on the pH and/or on the base of protein aggregation state: protein aggregation can be indeed induced lowering the detergent concentration, in this case from 0.03 to 0.007% of β-DM, mimicking protein clustering observed in thylakoid membranes, previously reported to be involved in activation of quenching mechanisms in LHC subunits, including LHCSR3^27,28,32,45–47^.

**Figure 5:**
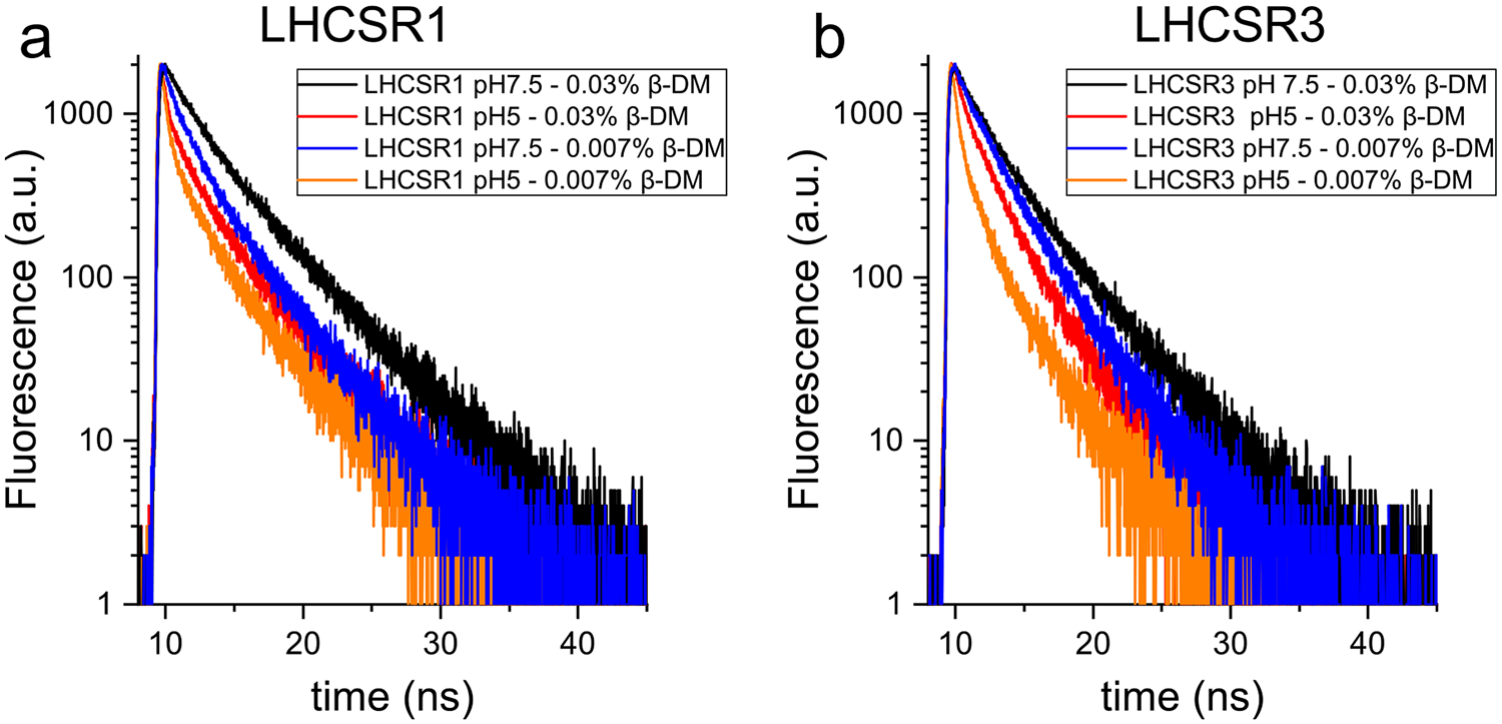
Time resolved fluorescence decay kinetics decay of reconstituted LHCSR1 and LHCSR3 recombinant proteins. LHCSR1 (**a**) and LHCSR3 (**b**) fluorescence decay kinetics were measured at 685 nm with excitation at 447 nm. The data herein reported are representative of two independent experiments. The figure was prepared by using OriginPro 2018 software https://www.originlab.com/.

As reported in Fig. 5 and in Supplementary Fig. S2, both LHCSR3 and LHCSR1 fluorescence decay kinetics were strongly dependent on detergent concentration and pH. In both cases, the longest fluorescence lifetimes were obtained at pH 7.5 and high detergent (Table 2). Lowering detergent concentration or pH caused, in either LHCSR1 or LHCSR3, a faster fluorescence decay kinetic, in agreement with previous finding on LHCSR3^27,28,32^. pH drop from 7.5 to 5 caused either at 0.03% or 0.007% a shorter fluorescence lifetime (Fig. 5 and in Supplementary Fig. S2), confirming the pH dependency of the quenching activity of these subunits. Comparing LHCSR1 and LHCSR3 proteins, the latter showed a faster decay compared in most of the conditions tested, with the shortest fluorescence lifetime being measured in the case of LHCSR3 at pH 5 and 0.007% β-DM. The pH dependent reduction of fluorescence lifetime was similar in percentage at 0.03% β-DM, but more pronounced in the case of LHCSR3 at 0.007% β-DM. The quenching activity induced by aggregation, obtained by reducing the detergent concentration from 0.03 to 0.007% was again more evident in LHCSR3 at pH 5 (Supplementary Fig. S2). These results suggest that the quenching activity of the two subunits is different, with LHCSR3 being the subunit that can reach the strongest quenching activity. Time resolved fluorescence was also measured at same pH and detergent concentration conditions also in the case of LHCII trimers, isolated from *C. reinhardtii* thylakoids (Supplementary Fig. S3). In this case the quenching induced by pH or aggregation was limited, if any, with only a ~ 10% reduction of average fluorescence lifetime in the case of pH 5 and 0.007% β-DM compared to the pH 7.5 and 0.03% β-DM condtion (Supplementary Fig. S3; Supplementary Table S1). This finding confirms the peculiar quenching properties of LHCSR subunits compared to other LHC complexes.

**Table 2:** Exponential fitting results of time-resolved fluorescence decay kinetics.

	τ_1_ (ns)	A_1_	τ_2_ (ns)	A_2_	τ_avg_ (ns)
LHCSR1 pH 7.5 0.03% β-DM	4.540 ± 0.020	0.509 ± 0.005	1.490 ± 0.030	0.491 ± 0.005	3.043 ± 0.029
LHCSR1 pH 7.5 0.007% β-DM	3.800 ± 0.002	0.357 ± 0.004	1.190 ± 0.002	0.643 ± 0.004	2.121 ± 0.016
LHCSR1 pH 5 0.03% β-DM	3.940 ± 0.020	0.331 ± 0.003	1.000 ± 0.020	0.669 ± 0.003	1.974 ± 0.018
LHCSR1 pH 5 0.007% β-DM	3.760 ± 0.030	0.225 ± 0.002	0.802 ± 0.020	0.775 ± 0.002	1.467 ± 0.018
LHCSR3 pH 7.5 0.03% β-DM	4.050 ± 0.030	0.474 ± 0.018	1.530 ± 0.030	0.526 ± 0.018	2.725 ± 0.080
LHCSR3 pH 7.5 0.007% β-DM	3.200 ± 0.020	0.520 ± 0.018	1.250 ± 0.030	0.480 ± 0.018	2.265 ± 0.065
LHCSR3 pH 5 0.03% β-DM	2.930 ± 0.020	0.372 ± 0.004	0.900 ± 0.020	0.628 ± 0.004	1.655 ± 0.019
LHCSR3 pH 5 0.007% β-DM	2.870 ± 0.020	0.161 ± 0.001	0.583 ± 0.010	0.839 ± 0.001	0.950 ± 0.010

Fluorescence kinetics reported in Fig. 5 were fitted with two exponential functions: decay constants (τ_1−2_) and amplitude (A_1−2_) of each component are reported in the table. Average fluorescence lifetimes were calculated as ΣA_i_τ_i_/ΣA_i_. Errors are reported as standard deviation (n = 2).

## Discussion

In the model organism for green algae, *C. reinhardtii*, LHCSR1 and LHCSR3 proteins were reported to be the main actors in the NPQ mechanism. Previous works demonstrated that *C. reinhardtii npq4* mutant, which lacks both genes encoding for LHCSR3 protein, was characterized by a reduced NPQ phenotype, with the residual NPQ dependent on LHCSR1 subunit^18,27^. Indeed, the *C. reinhardtii* double mutant *npq4 lhcsr1*, disrupted in all genes that encode for both LHCSR3 and LHCSR1 proteins, showed a null NPQ phenotype with high amounts of ROS formation and reduced growth rate^10,27,29^. Transcriptional analysis of *C. reinhardtii* cultures demonstrated that CO_2_ concentration influences the expression of *lhcSR1* and *lhcSR3* (*lhcSR3.1* and *lhcSR3.2*) genes, which are respectively transcriptionally activated and downregulated at high CO_2_ conditions^21,48^. This transcriptional control has been then validated by biochemical and immunoblotting analysis of *C. reinhardtii* cells grown at different CO2 concentrations: LHCSR3 is thus mainly accumulated in HL and low CO_2_ conditions, while LHCSR1 requires both HL and high CO_2_ to be expressed^49^. In this work functional and biochemical properties of LHCSR1 were analyzed and compared to LHCSR3 upon apoprotein overexpression in bacteria and in vitro refolding in presence of pigments. As in the case LHCSR3, LHCSR1 holoproteins were characterized by a very low amount of Chl b bound, confirming that LHCSR proteins have higher affinity for Chl a^24^. Both LHCSR1 and LHCSR3 were found to bind lutein and violaxanthin, with traces of neoxanthin being found in both holocomplexes. Pigment stoichiometry based on a putative number of 8 Chl bound per apoprotein^25^ revealed the presence of more than two carotenoids in LHCSR1, as previously reported for LHCSR3^24^. However, while a similar content of lutein and violaxanthin was detected in LHCSR3, a higher affinity for lutein was evident in the case of LHCSR1 (Table 1). In order to investigate a possible different composition of carotenoid binding sites in LHCSR1 compared to LHCSR3, deconvolution of the absorption spectra in the Soret region of both refolded proteins was performed to resolve the different absorption forms of Chl and carotenoids bound^40^. In both LHCSR1 and LHCSR3 three carotenoids binding sites were identified, consistently with the pigment stoichiometry proposed (Table 1), on the base of the absorption shift induced to the carotenoid absorption forms (Fig. 4). Inner sites L1 and L2 and a third more peripheral N1/V1-like site could be identified in both LHCSR1 and LHCSR3. Carotenoid binding site inducing the smaller absorption shift (7.5–8.1 nm) was identified in both LHCSR1 and LHCSR3 being occupied by violaxanthin and neoxanthin. The carotenoid binding site inducing an intermediate shift (11–13.4 nm), identified as L1, was mainly occupied by lutein in both complexes, while the carotenoid binding site inducing the strongest red shift (16–18.4 nm), identified as L2, was respectively preferentially occupied by lutein and violaxanthin in LHCSR1 and LHCSR3. Different occupancy of L2 site in LHCSR1 compared to LHCSR3 with a higher affinity for lutein is consistent with the results obtained by HPLC analysis of pigment extracts (Table 1). Interestingly, the structural model built for LHCSR1 and LHCSR3 (Fig. 2) revealed a different protein environment for one of the terminal ring of carotenoids in L2, comparing the two subunits: in the case of LHCSR1 the side chain of a phenylalanine (F116) residue was predicted to be orientated toward carotenoid in L2, possibly influencing the occupancy of this carotenoid binding site. This different L2 occupancy could be at the base of the different activity of the protein, influencing protein conformation and/or dynamics of protein activation toward a quenched state. In the case of LHCSR3 in vitro mutagenesis analysis demonstrated the involvement of both Chl 613, located close to carotenoid in L1, and Chl 603, located close to carotenoid in L2, in LHCSR3 quenching mechanisms^32^: a different composition of L2 in LHCSR1 could thus influence its quenching activity. Quenching properties of the two proteins have been analyzed by time resolved fluorescence analysis. LHCSR3 have been reported to be activated as a quencher by low pH, as in the case of lumen acidification upon photooxidative stress, and/or by protein aggregation. The latter condition has been previously reported to generally induce LHC protein to a quenching conformation and it has been linked to the in vivo clustering of antenna proteins observed in the thylakoid membranes^27,28,47,50,51^. The results obtained demonstrate that both proteins have a quenching activity strongly induced by either low pH or protein aggregation, showing the shortest lifetime at low detergent concentration and low pH. Consistently with NPQ observation in vivo in presence of LHCSR1 or LHCSR3 only^16^, both LHCSR subunits can be activated as quenchers at low pH to a much stronger extent compared to LHCII complexes, the major antenna subunits of PSII (Supplementary Fig. S3). However, LHCSR3 resulted to be more effective as a quencher compared to LHCSR1 showing the shortest fluorescence lifetime at low pH and low detergent concentration (Fig. 5, Table 2). It is important to note that LHCSR3 was also the subunit with the strongest reduction in percentage of its fluorescence lifetime comparing the conditions inducing the longest (pH 7.5 and 0.03% β-DM) and shortest (pH 5 and 0.007% β-DM) fluorescence lifetime (Supplementary Fig. S2), demonstrating a more efficient transition toward a quenched state. This result suggests that LHCSR3 is a better quencher than LHCSR1 during NPQ induction, providing a possible explanation for the evolutionary preference of LHCSR3 dependent quenching mechanisms in *C. reinhardtii* cells exposed to the most dangerous photooxidative conditions: high light and low CO_2_ concentration^21,49^. In this condition the high excitation pressure at the level of the photosynthetic apparatus is further aggravated by the low availability of CO_2_ the final acceptor of electrons coming from the photosynthetic electron transport chain. Indeed, at low CO_2_ concentration the metabolic flux through the carbon fixation reactions is reduced, slowing down the rate of ADP and NADP^+^ regeneration, further reducing the availability of these cofactors required to de-saturate the photosynthetic apparatus. Preferential expression of LHCSR3 compared to LHCSR1 in this condition can thus have the rationale of ensuring the highest quenching efficiency to prevent ROS formation. At high CO_2_ concentration, the risk of photoinhibition is reduced, while an higher fraction of excitation energy should be used to provide energy for carbon fixation: in this condition LHCSR3 is substituted with the less efficient LHCSR1 to meet the desired balance between photoprotection and photosynthetic efficiency. Further experimental work is required to prove this evolutionary perspective. Moreover, several details of the LHCSR1 dependent quenching need to be investigated, as the intramolecular quenching species formed^28^ or the interactions with other LHC subunits required for the NPQ activation^16,52^.

In conclusion, the in vitro analysis of LHCSR1 and LHCSR3 subunits suggested that both proteins can be activated as quenchers by reducing the pH of the medium and/or by inducing protein aggregation. However, LHCSR3 can reach a stronger quenching state compared to LHCSR1: this intrinsic properties of LHCSR1 and LHCSR3 are likely at the base of the different activity and regulation of LHCSRs subunit in vivo during NPQ induction. These results could pave the way for smart design of light harvesting systems for a proper balance of light harvesting and photoprotection.

## Methods

### LHCSR1 and LHCSR3 structure modeling

LHCSR1 and LHCSR3 protein structures were obtained using homology modeling techniques with the on-line tool SWISS-MODEL (https://swissmodel.expasy.org/)^53^. The model with the best GMQE (Global Model Quality Estimation) was selected for further analysis. Molecular graphics was performed with UCSF Chimera, developed by the Resource for Biocomputing, Visualization, and Informatics at the University of California, San Francisco, with support from NIH P41-GM103311^54^.

### Cloning of lhcSR1 and recombinant protein expression in *E. coli*

Coding sequence (CDS) of *lhcSR1* gene from *C. reinhardtii* was cloned in pET28a(+) expression vector, optimized for heterologous expression in *E. coli*. For this aim NcoI and HindIII restriction enzymes were used and CDS of *lhcSR1* was deprived of the first 16 amino acids, which are predicted to correspond to the chloroplast transit peptide.

The tagged version of the vector was created by amplification of the fragment of interest with following primers: ATATACCATGGGACGCTCGGTG (forward) and CACCAAAGCTTGATGGCCTTGAGGTTGTCGGG (reverse). The reverse primer was designed to remove the stop codon and create a sequence in frame with His-tag, contained in the vector. Amplified *lhcSR1* CDS sequence was confirmed by sequencing. Expression vector carrying *LHCSR1* CDS sequence were used to transform by electroporation BL21 *E. coli* competent cells. Transformed colonies were selected on plates for the acquired antibiotic resistance present in the pET28a(+) vector (kanamycin). Presence of the plasmid in the putative transformants was confirmed through amplification of the fragment of interest by colony PCR. LHCSR protein expression was induced by using IPTG 2 mM for 8 h at 37 °C and apoproteins were purified as inclusion bodies. LHCSR3 apoprotein was overexpressed in BL21 *E. coli* cells as previously reported^27^. SDS-PAGE was performed as described in^55^. Western blot analysis was performed using specific antibodies α-LHCSR1 and α-LHCSR3 (Agrisera, Sweden).

### Holoproteins in vitro refolding

LHCSR1 and LHCSR3 recombinant proteins were refolded in vitro as described in^27^ by adding isolated pigments from spinach^56^. Purification of tagged-reconstituted proteins was achieved by chromatography, using an affinity column with Nickel ions immobilized (IMAC), which specifically bind the His-tag^57^, followed by a further purification step by ultracentrifugation in sucrose gradient (0.1–1 M sucrose, Hepes pH 7.5 20 mM, 0.03% n-Dodecyl β-d-maltoside, herein named β-DM).

### Absorption and fluorescence steady state measurements

Absorption measurements were performed in the 350–750 nm region with a Cary 4000, Varian spectrophotometer. Steady state fluorescence measurements were performed with BeamBio custom device equipped with USB2000+OceanOptics spectrometer and custom LED light sources for excitation as reported in^32^. Deconvolution of absorption spectra in the Soret region was performed as reported in^40^. In particular, Chl a and b, lutein, violaxanthin and neoxanthin absorption forms were summed, applying different shifts, in order to reproduce the absorption spectra of both LHCSR1 and LHCSR3.

### Pigments analysis

Chl and carotenoids content were analyzed by high-performance liquid chromatography upon pigment extraction in 80% acetone according to^58^.

### Isolation of LHCII trimers

*Chlamydomonas reinhardtii* 4A + strain obtained by Chlamydomonas resource center (www.chlamyconnection.org) was grown in TAP medium at 70 μmol m^−2^ s^−1^. Thylakoid membrane were isolated from *C. reinhardtii* cells as previously described^59^. Isolated thylakoids were solubilized with 0.8% β-DM and ultracentrifuged in 0.1–1 M sucrsose gradient as previously reported in order to separate the different pigment binding complexes and purify LHCII complexes^38^. Pigment analysis of isolated LHCII trimers are reported in Supplementary Table S2.

### Time resolved fluorescence analysis

Time resolved fluorescence analysis was performed with a Chronos BH ISS Photon Counting instrument with picosecond laser excitation at 447 nm operating at 50 MHz. Fluorescence emissions were recorded at 685 nm with 4 nm bandwidth. Laser power was kept below 0.1 μW. Time resolved fluorescence kinetics of recombinant LHCSR complexes were measured at both pH 7.5 and pH 5 in high (0.03% β-DM) or low (0.007% β-DM) detergent concentration.

## Supplementary Information

Supplementary Information

## Data Availability

The datasets generated during and/or analyzed during the current study are available from the corresponding author on reasonable request.
