# Supplementary material for: Chlamydomonas reinhardtii LHCSR1 and LHCSR3 proteins involved in photoprotective non-photochemical quenching have different quenching efficiency and different carotenoid affinity
Source: Sci Rep. 2020 Dec 15;10:21957. doi: 10.1038/s41598-020-78985-w (PMC7738518; doi:10.1038/s41598-020-78985-w)
Supplement: Supplementary file 1 — Supplementary Information [file 41598_2020_78985_MOESM1_ESM.pdf]

SUPPLEMENTARY INFORMATION

Chlamydomonas reinhardtii LHCSR1 and LHCSR3 proteins involved in photoprotective non-photochemical quenching have different quenching efficiency and different carotenoid affinity

Federico Perozeni[1a], Giorgia Beghini[1a], Stefano Cazzaniga[1], Matteo Ballottari[1*]

[1] *Department of Biotechnology, University of Verona, Strada le Grazie 15, 37134 Verona, Italy*

[a] *The authors contributed equally to the work*

**Corresponding author, email: matteo.ballottari@univr.it , tel: +39 0458027807*

Figure S1. SDS-PAGE and immunoblotting analysis of recombinant LHCSR1 and LHCSR3 apoproteins. LHCSR apoproteins were purified from *Escherichia coli* as inclusion bodies: SDS-PAGE (a) and western blot (b) analysis were performed to investigate protein concentration and composition of the purified inclusion bodies. Different dilutions of another LHC protein (CP26 from *Arabidopsis thaliana*) previously quantified were loaded as a standard for a raw protein quantification by densitometry. Panel a: three dilutions of LHCSR1 (1:100, 1:50, 1:25), three dilutions of LHCSR3 (1:150, 1:100, 1:50) and three amounts of recombinant CP26 (0.88 ug, 1.056 ug and 1.76 ug) were loaded on SDS-PAGE gel. Gel staining was performed with Coomassie Blu R stain. Panel b: immunoblotting assay performed with antibody α-LHCSR1 and α-LHCSR3. The immunoblotting results shown in Panel b were obtained with samples (recombinant LHSR1 and LHCSR3 proteins) loaded on the same SDS-PAGE gel, blotted on nitrocellulose filter and then cut in order to develop the two different filter portions with the specific antibodies α-LHCSR1 and α-LHCSR3. The data herein reported are representative of two independent experiments.

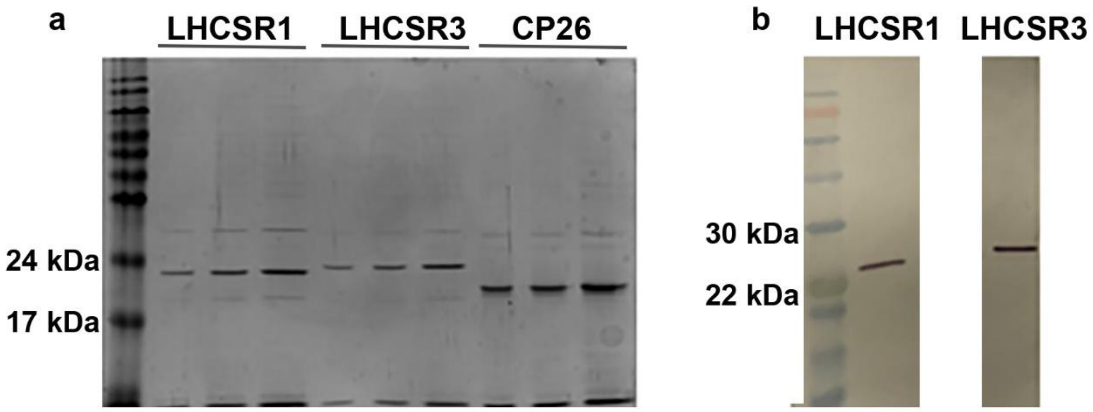

Figure S2. pH and/or detergent concentration dependent shortening of fluorescence lifetime in LHCSR1 and LHCSR3. Fluorescence lifetime of LHCSR1 and LHCSR3 reported in Table 2 were used to calculate the effect of reduced detergent concentration and/or pH reduction on the quenching properties of the two holoproteins. The data are reported as the ratio of fluoresce lifetimes measured respectively at 0.007% and 0.03% β-DM ($\tau_{avg\ 0.007\%}$ / $\tau_{avg\ 0.03\%,}$ aggregation effect) or at pH5 and pH 7.5 ($\tau_{avg\ pH\ 5}$/ $\tau_{avg\ pH\ 7.5,}$ pH effect) or at pH 5 + 0.007% β-DM and pH 7.5 + 0.03% β-DM ($\tau_{avg\ 0.007\%\ pH5}$ / $\tau_{avg\ 0.03\%\ pH\ 7.5,}$aggregation +pH). Error bars are reported as standard deviation (n=2). The figure was prepared by using OriginPro 2018 software https://www.originlab.com/ .

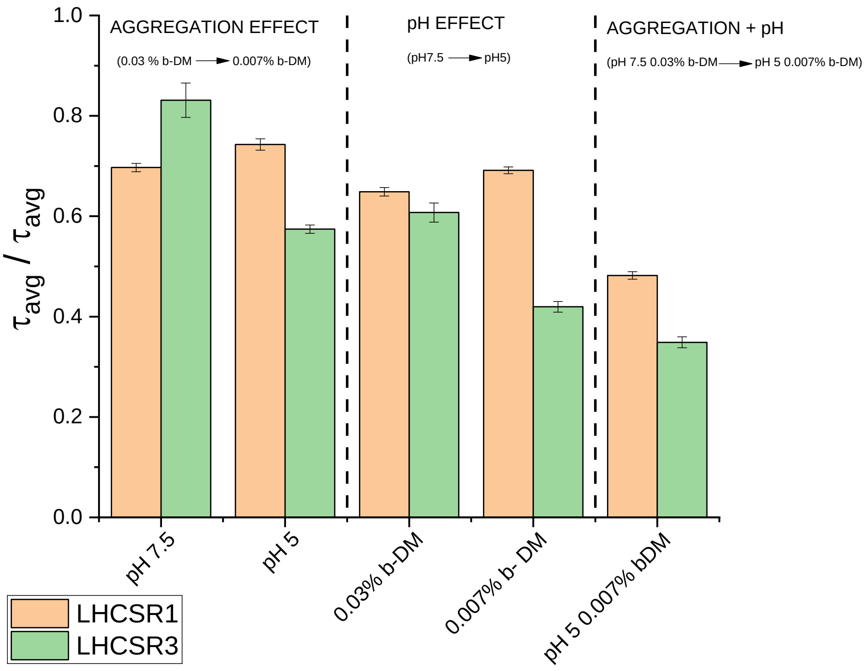

Figure S3. pH and/or detergent concentration dependent shortening of fluorescence lifetime in LHCII compared to LHCSR subunits. Fluorescence lifetime of LHCII trimers isolated from *C. reinhardtii* thylakoid membranes were measured by TCSPC analysis at different pH (7.5 or 5) and detergent concentration (0.03% or 0.007% β-DM). (A) fluorescence decay kinetics; (B) average fluorescence lifetimes calculated as described in Table S3 are reported in (B) compared to the LHCSR1 and LHCSR3 case. Error bars are reported as standard deviation (n=2). The figure was prepared by using OriginPro 2018 software https://www.originlab.com/

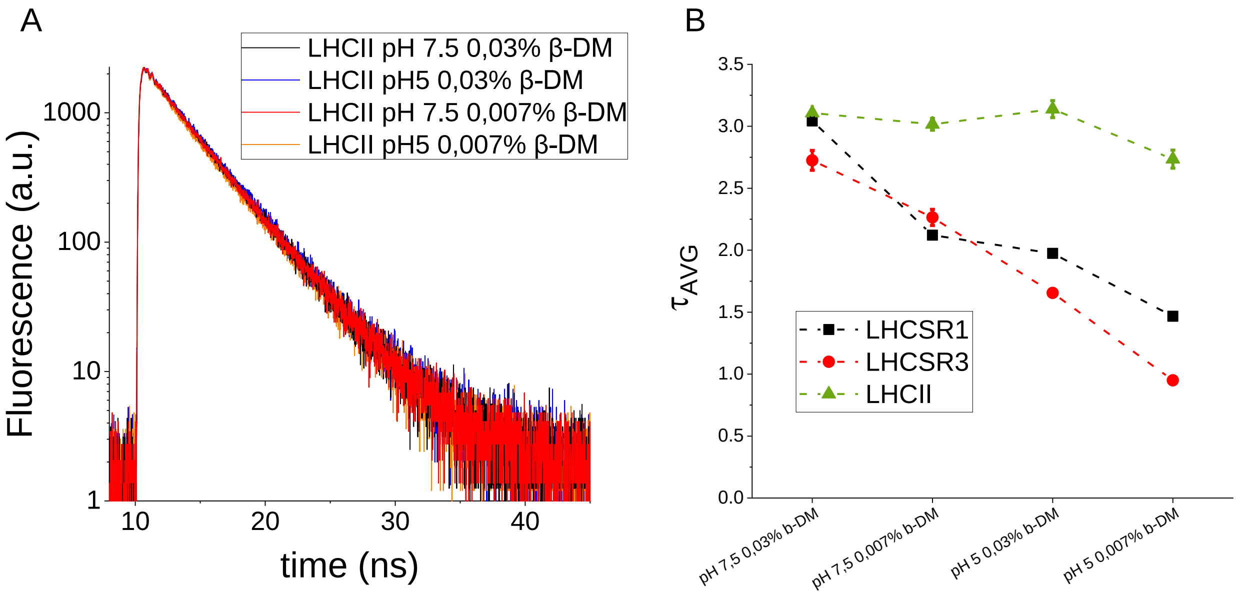

Table S1. Exponential fitting results of time-resolved fluorescence decay kinetics in the case of LHCII samples. Fluorescence kinetics reported in Figure S3A were fitted with two exponential functions: decay constants ($\tau_{1\text{-}2}$) and amplitude ($A_{1\text{-}2}$) of each component are reported in the table. Average fluorescence lifetimes were calculated as $\Sigma A_i \tau_i / \Sigma A_i$. Errors are reported as standard deviation (n=2).

	**τ1 (ns)**	**A1**	**τ2 (ns)**	**A2**	**τavg (ns)**
**LHCII pH 7.5 0,03% β-DM**	3.430 ± 0.042	0.872 ± 0.020	0.914 ± 0.008	0.128 ± 0.020	3.108 ± 0.012
**LHCII pH 7.5 0,007% β-DM**	3.330 ± 0.028	0.876 ± 0.011	0.793 ± 0.048	0.124 ± 0.011	3.016 ± 0.048
**LHCII pH 5 0,03% β-DM**	3.375 ± 0.092	0.907 ± 0.005	0.831 ± 0.025	0.093 ± 0.005	3.139 ± 0.068
**LHCII pH 5 0,007% β-DM**	3.320 ± 0.028	0.771 ± 0.018	0.761 ± 0.021	0.229 ± 0.018	2.735 ± 0.072

Table S2. Pigment analysis of native isolated LHCII complexes purified from *C. reinhardtii* thyalkoids. Total amount of carotenoids (Cars) and the level of the different xanthophyll were normalized to 8 chlorophyll (Chl tot) content per aproprotein. Neo: neoxanthin; Vio: violaxanthin; Lut: lutein. Errors are below 6% in each case (n=3).

	**Chl a/Chl b**	**Chl/Car**	**Chl tot**	**Neo**	**Vio**	**Lut**	**Cars**	**Lut/Vio**
**LHCII**	1.46	3.85	14.00	1.01	0.90	1.60	3.51	1.78
